# Comparison of four synthetic CT generators for brain and prostate MR-only workflow in radiotherapy

**DOI:** 10.1186/s13014-023-02336-y

**Published:** 2023-09-05

**Authors:** Damien Autret, Camille Guillerminet, Alban Roussel, Erwan Cossec-Kerloc’h, Stéphane Dufreneix

**Affiliations:** 1 Institut de Cancérologie de l’Ouest, Angers, France; 2 CEA, List, Laboratoire National Henri Becquerel (LNE-LNHB), Palaiseau, France

**Keywords:** Synthetic CT, Deep learning method, Bulk density assignation, MR-only workflow

## Abstract

**Background:**

The interest in MR-only workflows is growing with the introduction of artificial intelligence in the synthetic CT generators converting MR images into CT images. The aim of this study was to evaluate several commercially available sCT generators for two anatomical localizations.

**Methods:**

Four sCT generators were evaluated: one based on the bulk density method and three based on deep learning methods. The comparison was performed on large patient cohorts (brain: 42 patients and pelvis: 52 patients). It included geometric accuracy with the evaluation of Hounsfield Units (HU) mean error (ME) for several structures like the body, bones and soft tissues. Dose evaluation included metrics like the D_mean_ ME for bone structures (skull or femoral heads), PTV and soft tissues (brain or bladder or rectum). A 1%/1 mm gamma analysis was also performed.

**Results:**

HU ME in the body were similar to those reported in the literature. D_mean_ ME were smaller than 2% for all structures. Mean gamma pass rate down to 78% were observed for the bulk density method in the brain. Performances of the bulk density generator were generally worse than the artificial intelligence generators for the brain but similar for the pelvis. None of the generators performed best in all the metrics studied.

**Conclusions:**

All four generators can be used in clinical practice to implement a MR-only workflow but the bulk density method clearly performed worst in the brain.

**Supplementary information:**

The online version contains supplementary material available at (10.1186/s13014-023-02336-y).

## Background

Interest is growing to use Magnetic Resonance Imaging (MRI) as the only imaging modality for radiotherapy planning to take advantage of its soft tissue contrast and remove inter-modality registration uncertainties [1]. However, MRI cannot be used directly for dose calculation because MR intensities correlate with proton densities and magnetic relaxation tissue properties whereas dose calculation in treatment planning systems requires electron densities of the tissues. Several methods exist to generate a synthetic Computed Tomography (sCT) from the MR images. The bulk density methods consist in segmenting the MR image and assign a Hounsfield Unit (HU) value to each voxel based on its corresponding tissue class. These methods are often associated to long MR acquisition time and limited by the weak and similar MR signal of bony structures and air [2]. The atlas-based methods are based on the registration of MR images with co-registered MRI-CT atlases. Their performances are affected by the ability of the atlas dataset to represent the clinical case [3]. Recently, artificial intelligence (AI) and more specifically deep learning methods have been used to generate sCT with an algorithm previously trained on MRI and CT images [4]. They showed promising results compared to atlas based methods in terms of Hounsfield Unit accuracy [5].

In 2018, two reviews were published to give an overview of the performances of sCT generated by the bulk density or the atlas-based methods [6, 7]. Similarly in 2021, two reviews were published to summarize common deep learning networks for sCT generation and evaluate their performances [8, 9]. However, these studies do not enable a direct comparison of different sCT generators because methodologies can differ and the evaluation is not performed on the same dataset. Numerous AI generators are also developed by research teams and are not commercially available [3].

The aim of this study was to evaluate 4 commercially available sCT generators: one based on the bulk density method and three based on deep learning methods. The comparison was performed on large patient cohorts for two anatomical localizations (brain: 42 patients and pelvis: 52 patients).

## Materials and methods

### Patient data, image acquisition and sCT generators

The study protocol was identical to the one described in [10] and approved by the Research Ethics Committee of the Hospital. A total of 94 patients were enrolled (42 treated for brain tumors and 52 for prostate cancers). Median age of the brain cohort was 66 years [35y–84y] with both gender equally represented. Median age of the pelvis cohort was 76 years [54y–89y]. Details on the patient cohorts are given in Table 1. Patients with implants generating MR artifacts were excluded from the study (1 brain and 5 pelvis).

**Table 1:** Target and prescribed dose for the brain and pelvis cohorts

Brain (n = 42)	Target	Meningioma	5
		Glioma	34
		Carcinopharyngioma	1
		Astrocytoma	2
	Dose (Gy)	60 − (30 × 2)	28
		54 − (30 × 1.8)	4
		52.2 − (29 × 1.8)	1
		50.4 − (28 × 1.8)	4
		40 − (15 × 2.67)	5
Pelvis (n = 52)	Target	Prostate alone	10
		Prostate and vesicles	5
		Prostate, vesicles and lymph nodes	30
		Prostate and bone metastases	3
		Prostate cavity and pelvis	3
		Pelvis	1
	Dose (Gy)	78 − (39 × 2)	46
		74 − (37 × 2)	1
		72 − (36 × 2)	2
		66 − (33 × 2)	1
		60 − (20 × 3)	2

A CT was first acquired with a Siemens Somatom Confidence RT scan (120 kV, 2 mm slice thickness) and patients were positioned in radiotherapy treatment conditions. Prostate patients were in a supine position with a knee support cushion. Brain patients were immobilized in a thermoplastic three-point mask. MR images were acquired in identical treatment conditions on a 1,5 T Siemens Magnetom Aera XJ MRI scan using a provided flat table. MRI scan parameters for each sequence are detailed in Additional file 1: Table S1. A mean time of 4 days (range 0–11) was observed between the CT and MR exams.

sCT images were generated by 4 commercially available solutions:

- The Syngo_BD generator (Siemens, Munich, Germany) is based on bulk density method. Based on various MRI sequences, tissues are categorized into five classes. For the pelvis cohort, each voxel is assigned a tissue class with its corresponding HU value generating sCT images among five discrete HU values. For the brain cohort, each voxel is assigned a probability of belonging to each of the classes. As a consequence, sCT images for brain showed a continuous HU spectrum ranging from − 1000 to 1096 HU. The Syngo_BD generator was evaluated for brain [10, 11] and pelvis [10, 12, 13].
- The MRI Planner software (Spectronic Medical AB, Helsingborg, Sweden) generated sCT images using the deep-learning based Transfer Function Estimation algorithm. It was evaluated in its previous version based on an automated atlas-based conversion method for brain [14] and pelvis [15].
- The neural network of the Syngo_AI architecture used by Siemens consists of a densely connected UNet associated to a conditional Generative Adversarial Network for sCT reconstruction. It was evaluated for brain in [16].
- The sCT model of Therapanacea was trained using end-to-end ensembled self-supervised GANs endowed with cycle consistency and sCT images were provided by the MR-Box by ART-Plan software (Therapanacea, Paris, France). To the best of the author’s knowledge, this generator has not been previously evaluated.

**Fig. 1:**
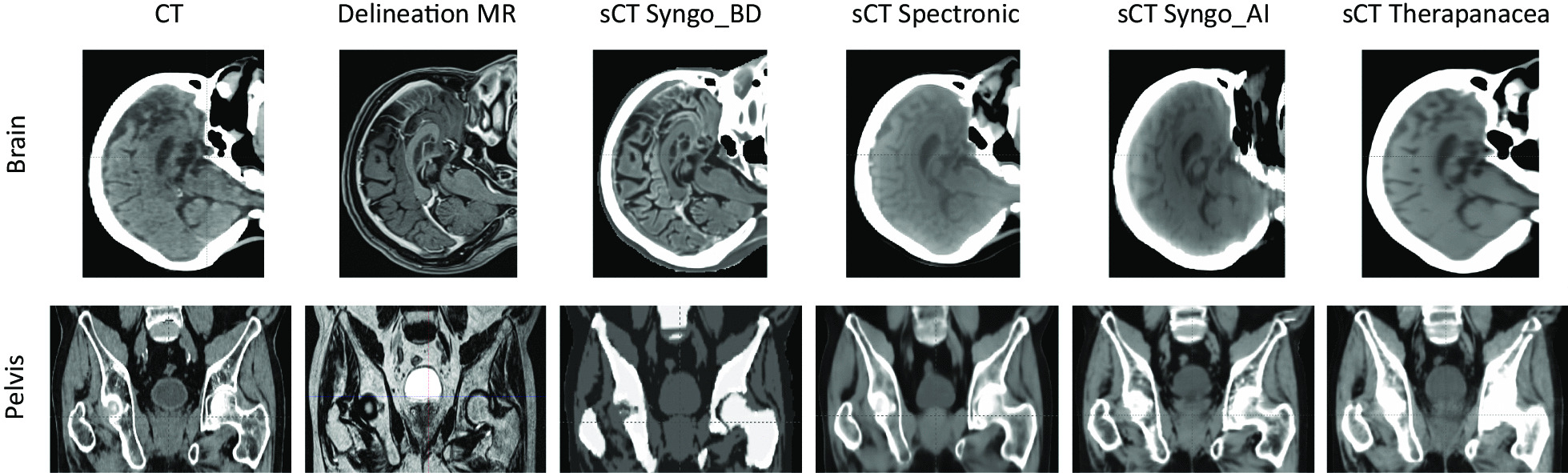
Upper row: example of sagittal images for the CT, MR and sCTs studied of the brain cohort (viewing window for CT and sCT: [ − 20; 100] HU). Lower row: frontal images for the CT, MR and sCTs studied of the pelvis cohort (viewing window for CT and sCT: [ − 125; 225] HU)

An example of sCT images generated by the four different solutions is shown in Fig. 1.

In the context of a MR-only workflow, at least one MR sequence is needed to delineate the PTV and OARs. Some of the solutions used the delineation MR sequence to generate a sCT whereas others required additional MR sequences. A summary of the MR sequences required for each solution is given in Table 2. Most of the additional MR sequences lengthen the exam of only a few minutes, except for the Syngo_BD generator in the brain cohort: MR sequences required for the sCT generation took approximatively 15 min which can be long and uncomfortable for the patient.

**Table 2:** Description of the MR sequences required for delineation and generation of the sCT

	Delineation	Syngo_BD	Spectronic	Syngo_AI	Therapanacea
Brain	*T1 Gd 3D*	T2 Space 3D T2 PETRA 3D (Bones) T1 VIBE DIXON 3D T2 FLASH Gradient Echo 2D (Vessels)	T1 VIBE DIXON 3D	T1 VIBE DIXON	*T1 Gd 3D*
Pelvis	*T2 Space 3D*	T1 VIBE DIXON 3D	*T2 Space 3D*	T1 VIBE DIXON	*T2 Space 3D*

Sequences similar to the delineation sequences are Italic

### Design of the study

**Fig. 2:**
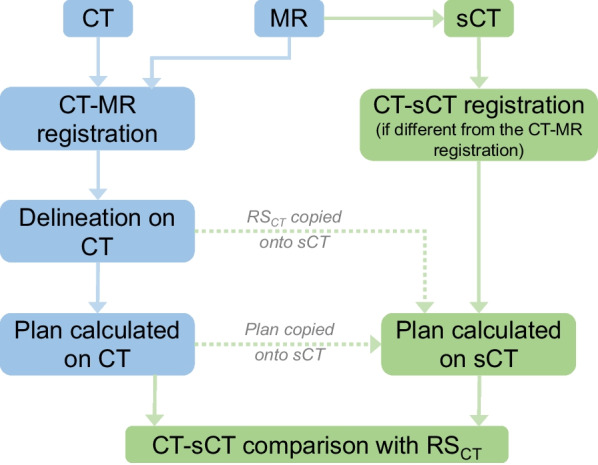
Design of the study. Blue: standard workflow. Green: MRI-only workflows for Brain and Pelvis. RS: Radiotherapy Structures

The study and standard workflows are described in Fig. 2. MR and CT were rigidly registered based on bony structures in the Aria environment (v15.1, Varian Medical Systems, Paolo Alto, USA). Delineation was performed on CT. The sCT shared the same spatial and temporal frame of reference as the MR sequence it was generated from. If the sCT was generated from the delineation MR sequence, the MR-CT registration could be used to register the sCT with the CT. If an additional MR sequence was needed to generate the sCT, a specific sCT-CT rigid registration was however required. During this study, delineation sequence and additional sequences did not share the same frame of reference due to the acquisition protocol. In more details:

- For the brain cohort, the MR-CT registration was used to register the sCT with the CT for the Therapanacea generator. A rigid registration between sCT and CT was performed for the other generators (Syngo_BD, Spectronic and Syngo_AI).
- For the pelvis cohort, the MR-CT registration was used to register the sCT with the CT for the Spectronic and Therapanacea generators. A rigid registration between sCT and CT was performed for the Syngo_BD and Syngo_AI generators

Structures were delineated on the CT and plans generated on the CT for treatment were copied and recalculated on the sCT. CT structures were duplicated on the sCT using the either the CT-MR registration or the sCT-CT registration depending on the MR sequence required to generate the sCT. A comparison in terms of Hounsfield Unit (HU) and dose between the CT and sCT was performed based on CT structures.

### Image comparison

A methodology identical to the one described in [10] was followed. A comparison was made between the HUs of the sCT and the HUs of the reference CT scan for various structures, all contoured on the CT. Body contours were generated by thresholding at − 350 HU, followed by a morphological hole-filling. Bone contours (skull for the brain, femoral heads for the pelvis) were generated by thresholding the respective images at 100 HU. For the brain patients, soft tissue contours were generated by thresholding the sCTs and CTs at -100 HU and subtracting the previously generated skull structure. For the pelvis patients, bladder and PTV_prostate_ were delineated on the CT and copied on the sCT.

Mean HU were extracted in each volume and differences were calculated between the CT and sCT for each patient and each generator. Mean Error (ME) and Mean Absolute Error (MAE) were calculated according to the formulas:

$$ME = \overline{HU}_{sCT} - \overline{HU}_{CT}$$

$$MAE = \left| {\overline{HU}_{sCT} - \overline{HU}_{CT} } \right|$$

where $\overline{HU }$ is the mean HU value of the considered structure.

### Dose comparison

Prostate patients could have up to 3 PTVs (prostate, prostate and vesicles, pelvis), each associated to a different plan. For each PTV, a 2-arcs (or 2-partial-arcs) VMAT plan was optimized and calculated within the Eclipse environment (Varian Medical Systems) based on the CT associated to the CT structures, as in clinical practice. Plans were then copied on the sCT associated. No re-optimization was performed and the MU values were left unchanged. Although most sCT generators (except Therapanacea) provide a specific CT calibration curve to be applied to the sCT, the same curve was used for converting HU into electron densities for the CT and all sCT. This choice was made to eliminate the influence of the CT calibration curve on the results. All plans were calculated at 6 MV with the AcurosXB algorithm and a 1.5 mm grid size.

Similarly to the methodology described in [10], a DVH analysis was conducted on the PTV and OAR structures by reporting Dmean, D2% and D98%. Mean errors and mean absolute errors were calculated and normalized to the corresponding dose metric on the CT. A comparison of the dose distributions in the axial slice crossing the isocenter was performed by calculating the mean dose difference and the 1%-1 mm global gamma index with a cut-off isodose of 2%. Gamma and DVH analysis were reported relatively to the prescribed dose.

## Results

### Image comparison

**Fig. 3:**
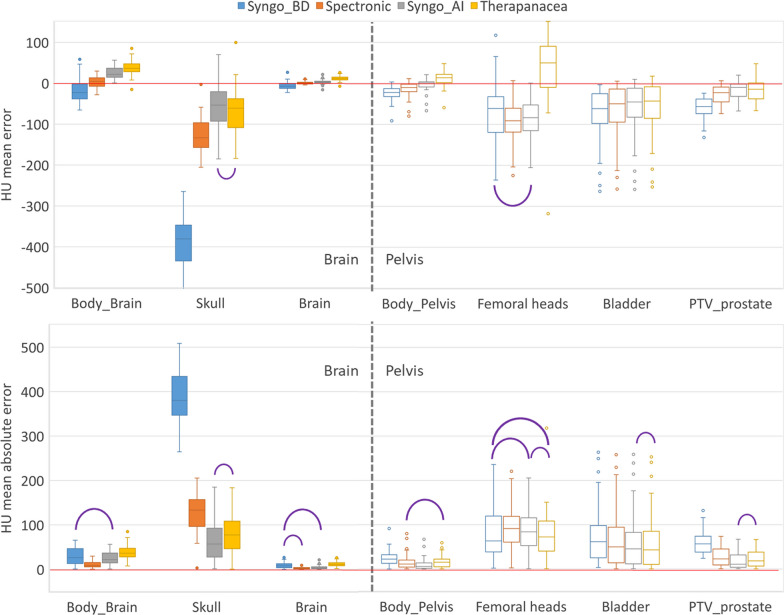
HU mean error (top) and mean absolute error (bottom) for several structures and four sCT generators. Purple semicircles link boxplots with a p-value smaller than 0.05 (Wilcoxon signed-rank test)

Boxplots of HU mean errors and mean absolute errors are shown in Fig. 3 for body, bone and soft tissue structures. For the brain, the largest difference between the generators was observed for the skull: the mean error is − 381 HU for Syngo_BD against − 127 HU for Spectronic and − 63 HU and − 72 HU for Syngo_AI and Therapanacea respectively. The large mean error for Syngo_BD was attributed to the fact that the maximum HU value of the sCT was limited to 1000 HU whereas values up to 3000 HU could be found on CT images. For the pelvis, although all 4 generators present the same mean absolute error for the femoral heads (around 78 HU), Therapanacea tended to overestimate the HU whereas the other generators tended to underestimate the HU.

The MAE of the body in the brain cohort was 30, 22, 25 and 40 HU respectively for Syngo_BD, Spectronic, Syngo_AI and Therapanacea generators. Similarly, the MAE of the body in the pelvis cohort was 21, 11, 7 and 17 HU respectively.

### Dose comparison

#### DVH metrics

**Fig. 4:**
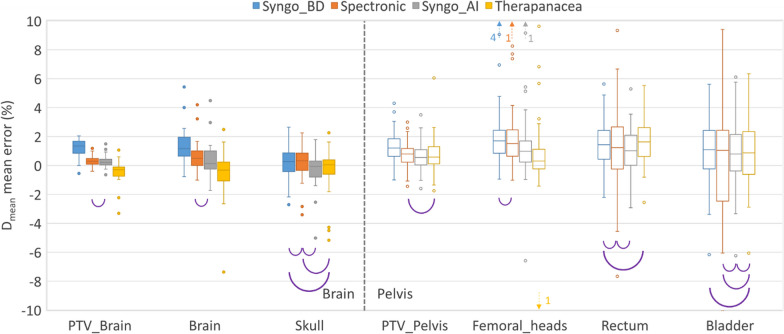
D_mean_ mean error for several structures and four sCT generators. Purple semicircles link boxplots with a p-value smaller than 0.05 (Wilcoxon signed-rank test)

Boxplots of the Dmean mean errors are shown in Fig. 4. Mean and standard deviations for D2% and D98% are given in Additional file 1: Table S2. All median values were below 2% but outliers higher than 10% were reported in the pelvis cohort. It was verified that the outliers were not associated to the same patient for all generators. Highest mean values were common for the Syngo_BD generator. In the brain cohort, Therapanacea was the only generator showing an underdosage on the sCT compared to the CT.

#### Gamma analysis

**Fig. 5:**
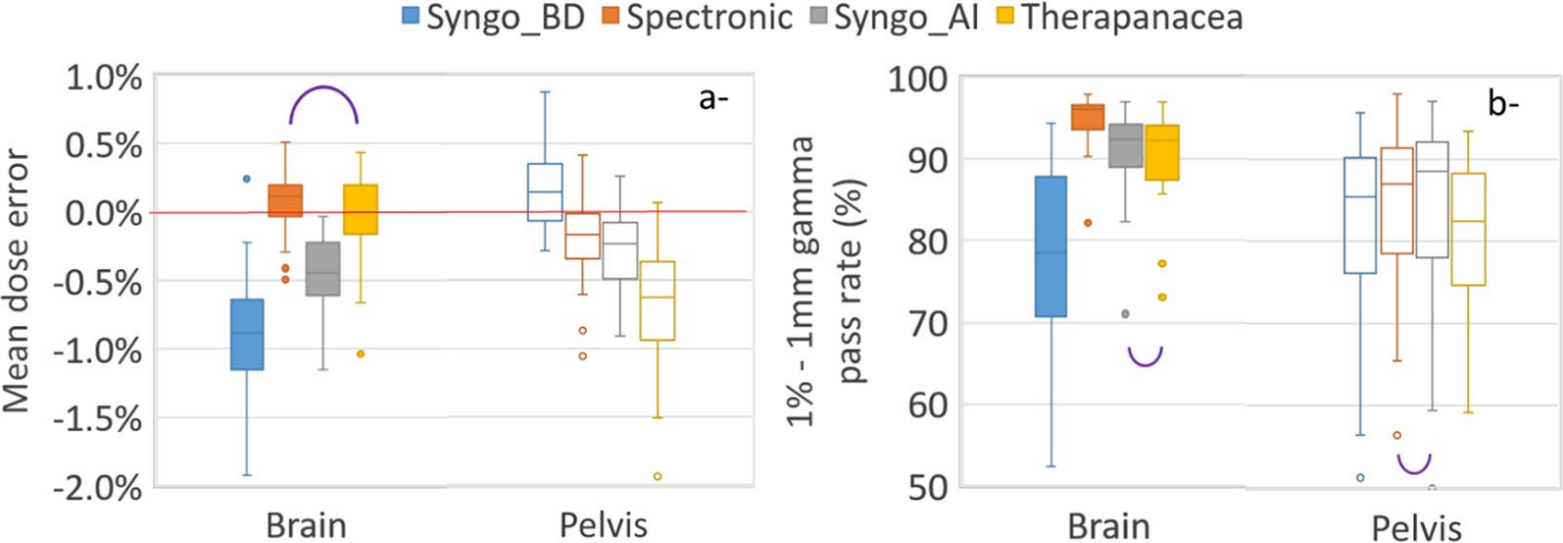
Mean dose error (**a**) and 1%-1 mm gamma pass rate (**b**) reported on the axial slice crossing the isocenter for the brain and pelvis cohorts. Purple semicircles link boxplots with a *p*-value smaller than 0.05 (Wilcoxon signed-rank test)

Boxplots of the mean dose difference and the 1%-1 mm gamma index in the axial slice crossing the isocenter are reported in Fig. 5. All dose differences were smaller than 2% and means were smaller than 1%. Therapanacea performed best in the brain cohort but worst in the pelvis cohort. For the brain cohort, the mean gamma pass rate for Syngo_BD (78%) was lower than those of the deep learning generators (95%, 91% and 91% for Spectronic, Syngo_AI and Therapanacea respectively). For the pelvis cohort, mean gamma pass rate were 82%, 85%, 84% and 81% respectively for Syngo_BD, Spectronic, Syngo_AI and Therapanacea.

## Discussion

This study evaluated several sCT generators based on bulk density generator or deep learning generators. All sCT images were evaluated on the same patient cohort and using the same metrics. MAE of the body in the brain cohort ranged from 22 to 44 HU and from 7 to 21 HU for the body in the pelvis cohort. Comparison with the literature was voluntary not performed because the MAE in this study was defined as an overall metric which differ from the one usually evaluated by other authors (voxel wise approach) [6–9]. Only MAE within this study can be compared without bias. All median DVH metrics were below 2% in agreement with the literature [8]. Some outliers revealed deviations higher than 10%, especially for the femoral heads. However these deviations were expressed relative to the corresponding metric on the CT and would be minimized if reported against the prescription dose. For example, the D_mean_ ME for the femoral heads ranged from − 15.2 to 9.6% if normalized to the D_mean_ on the CT for the Therapanacea generator (Fig. 4) but from − 1.4 to 1.5% if normalized to the prescribed dose. Similar improvement of the results could be achieved for the rectum and bladder: no outlier higher than 5% was observed if normalized to the prescribed dose. Gamma pass rates of the deep learning generators were above those reported in the literature (89% for deep learning generators [8]) for the brain cohort but slightly lower for the pelvis cohort. Overall, results found in this study were in agreement with data available in the review papers on sCT [6–9] or even better suggesting a constant evolution of the generators. Similar results were also reported in a study evaluating the feasibility of an MR-only prostate radiotherapy workflow with an automated atlas-based conversion method [17] confirming the clinical validation of sCTs. The comparison with literature is however limited by the heterogeneity of metrics that can be found, as stressed previously for the MAE. Similar issues can be found for other metrics, like for example the normalization performed during dose evaluation. The strength of our study is that all sCT generators were evaluated on the same cohort with the same metrics, allowing for a direct comparison between the sCT generators under standardized conditions.

A possible bias in the dose evaluation is the CT calibration curve used for converting HU into electron densities for sCT. In this study, the same CT calibration curve was applied for all sCTs but most generators (except Therapanacea) provide a specific curve to be applied to the sCT. O’Connor et al. [12] showed that modifying the relative electron and mass density curve applied to the sCT could improve the average percentage dose difference of 1.1%. Results given in Fig. 4 could thus potentially be slightly improved.

It is interesting to note that large difference in terms of HU ME were not correlated with large differences in terms of dose ME (see for example the Skull or Femoral heads structures in Figs. 3 and 4). In the pelvis cohort, high D_mean_ ME and low gamma pass rates were identified and were attributed to the methodology used: only a rigid registration was performed between the sCTs and CT which is not adequate because of variations of rectum and bladder filling between the MR and CT exams. This does not affect the comparison between the generators but results could have been improved by using a deformable registration.

Performances of the bulk density generator (Syngo_BD) were generally worse than the artificial intelligence generators for the brain cohort. Similar results were found for the pelvis cohort in terms of HU and dose metrics for all generators even if the sCT visual aspect of the Syngo_BD generator was clearly worse than others (Fig. 1). Statistical correlations evaluated with a Wilcoxon signed-rank test were found between all sCT generators depending on the metric studied. The most frequent correlation was observed between the Spectronic and Syngo_AI generators for the DVH metrics (Fig. 4). It is difficult to designate one generator better than the others because none of them performed best in all HU ME, dose ME and gamma evaluations. For example, Spectronic performed best regarding the gamma pass rate and mean dose error in the brain (Fig. 5) but performed worst regarding the HU ME for the skull (Fig. 3). The Therapanacea generator, which has not yet been evaluated to the authors’ knowledge, showed similar performances to the other artificial intelligence methods. A summary of the advantages and disadvantages of each sCT generator is given in Table 3.

**Table 3:** Advantages and disadvantages of the sCT generators investigated in this study

	Advantages	Disadvantages	Others
Syngo_BD		Brain: HU underestimation for the skull Brain: overall worst dosimetric results (D_mean_ ME and gamma analysis) Pelvis: unrealistic femur reconstruction (see [10]) Brain and pelvis: requires an additional registration between MR sequences	Pelvis: discrete HU values Brain: max 1000 HU on the sCT image
Spectronic	Pelvis: sCT generated from the delineation MR sequence Brain: overall smallest D_mean_ ME Brain and pelvis: overall best gamma analysis	Brain: requires an additional registration between MR sequences	Pelvis: the generator automatically fills the rectum to avoid taking into account unreproducible gas when preparing the treatment plan Brain: the generator does not generate a sCT if high density materials like dental appliance artefact the MR image
Syngo_AI	Brain: overall smallest D_mean_ ME	Brain and pelvis: requires an additional registration between MR sequences	
Therapanacea	Brain and pelvis: sCT generated from the delineation MR sequence		No CT calibration curve provided Pelvis: only sCT to overestimate the HU of the femoral heads

Others aspects have to be considered in the context of a MR-only workflow: for the pelvis cohort, the delineation MR sequence should cover the entire field of view and not be limited to the prostate region as can be performed currently in a CT-based workflow. The MR sequence used by the generator is also of importance. Some generators require sequences differing from the one used for delineation (Table 1) which can lengthen the MR exam. The anatomy of the patient between the sequences can also change (bladder and rectum filling) and affect the analysis. The comparison can also be affected by variations (anatomical or signal-to-noise ratio for example) between the MR sequences. The validation of a MR-only workflow should include the evaluation of patient positioning on the linac. Such evaluation should be performed for each sCT generator/imaging device combination available. Some studies showed that a 2 mm accuracy compared to a CT positioning could be achieved [16, 17].

The limitations of this study include the retrospective analysis and the lack of statistical analysis. The choice of the evaluation metrics could also be further discussed because a large variety of metrics and definition of metrics can be observed in the literature [8, 9]. Future work will include the implementation of an MR workflow in our department and will focus on the quality assurance of the sCT. This crucial step is required to ensure the sCT generated will not introduce a treatment error and metrics for the validation of a sCT without a CT will be needed.

## Conclusions

This study evaluated four commercially available sCT generators for a MR-only workflow in radiotherapy using a large patient cohort (42 primary brain tumors and 52 prostate cancer). Evaluation was performed in terms of intensity-based metrics accuracy and dose evaluation and result in agreement with the literature were reported. All four generators can be used in clinical practice to implement a MR-only workflow but the bulk density method clearly performed worst in the brain.

### Supplementary information

**Additional file 1. Table S1:** MRI scan parameters for the segmentation sequences (black) and sCT specific sequences (blue). BW: band width; ET: Echo time; TR: repetition time; FA: flip angle; ST: slice thickness; FOV: field of view. **Table S2:** mean value of the mean error for Dmean, D2% and D98% for various structures in the brain and pelvis cohorts. Standard deviation are given in parenthesis.

## Data Availability

The datasets used and/or analysed during the current study are available from the corresponding author on reasonable request.
