# Supplementary material for: Comparison of four synthetic CT generators for brain and prostate MR-only workflow in radiotherapy
Source: Radiat Oncol. 2023 Sep 5;18:146. doi: 10.1186/s13014-023-02336-y (PMC10478301; doi:10.1186/s13014-023-02336-y)
Supplement: Supplementary file 1 — Additional file 1. Table S1: MRI scan parameters for the segmentation sequences (black) and sCT specific sequences (blue). BW: band width; ET: Echo time; TR: repetition time; FA: flip angle; ST: slice thickness; FOV: field of view. Table S2: mean value of the mean error for Dmean, D2% and D98% for various structures in the brain and pelvis cohorts. Standard deviation are given in parenthesis. [file 13014_2023_2336_MOESM1_ESM.docx]

Supplemental Table 1: MRI scan parameters for the segmentation sequences (black) and sCT specific sequences (blue). BW: band width; ET: Echo time; TR: repetition time; FA: flip angle; ST: slice thickness; FOV: field of view


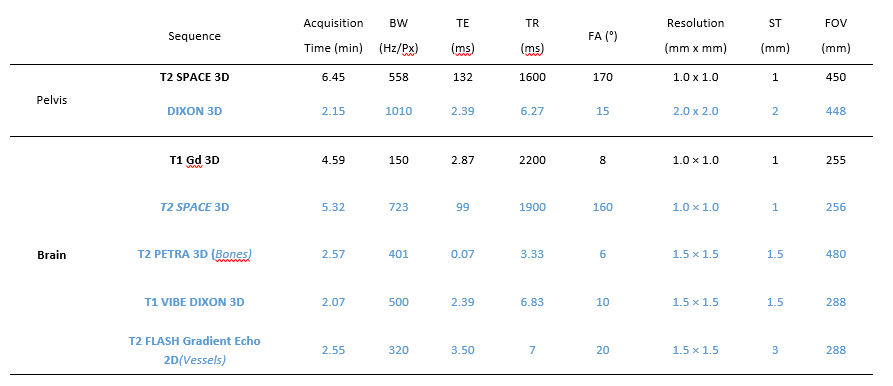


Supplementary Table 2: mean value of the mean error for D_mean_, D_2%_ and D_98%_ for various structures in the brain and pelvis cohorts. Standard deviation are given in parenthesis.

|  |  | Brain | | | Pelvis | | | |
| --- | --- | --- | --- | --- | --- | --- | --- | --- |
|  |  | PTV | Brain | Skull | PTV | Femoral heads | Rectum | Bladder |
| D_mean_ | Syngo_BD | 1.2 (0.6) | 1.4 (1.1) | 0.1 (1.2) | 1.3 (1.1) | 2.6 (3.7) | 1.5 (1.6) | 1.1 (2.3) |
|  | Spectronic | 0.3 (0.3) | 0.6 (1.0) | 0.0 (1.2) | 0.8 (0.9) | 2.1 (3.3) | 1.2 (3.0) | 0.3 (4.0) |
|  | Syngo_AI | 0.3 (0.4) | 0.3 (1.1) | -0.3 (1.1) | 0.6 (1.0) | 1.7 (7.1) | 1.1 (1.6) | 0.8 (2.4) |
|  | Therapanacea | -0.5 (0.7) | -0.5 (1.5) | -0.3 (1.5) | 0.8 (1.2) | 0.5 (6.1) | 1.7 (1.7) | 0.9 (2.3) |
| D_2%_ | Syngo_BD | 1.7 (0.7) | 1.4 (0.5) | 1.3 (1.2) | 1.9 (1.7) | 2.3 (3.0) | 1.3 (2.0) | 1.6 (1.6) |
|  | Spectronic | 0.4 (0.5) | 0.4 (0.8) | -1.2 (2.3) | 1.1 (1.1) | 1.4 (2.5) | 1.1 (1.3) | 1.2 (1.2) |
|  | Syngo_AI | 0.6 (0.7) | 0.3 (0.4) | 0.2 (0.9) | 1.0 (1.6) | 1.2 (2.8) | 0.8 (1.2) | 1.1 (1.5) |
|  | Therapanacea | -0.2 (0.5) | -0.4 (0.5) | -0.2 (0.9) | 1.2 (1.5) | 1.4 (3.0) | 1.4 (1.4) | 1.5 (1.4) |
| D_98%_ | Syngo_BD | -0.1 (2.7) | -3.1 (3.7) | -1.9 (3.1) | 0.3 (2.9) | -0.5 (8.1) | 12.1 (72.5) | 3.1 (29.6) |
|  | Spectronic | 0.3 (0.5) | 0.4 (1.8) | 0.5 (1.8) | 0.1 (1.7) | -1.9 (13.0) | 11.7 (74.0) | 2.6 (30.4) |
|  | Syngo_AI | 0.1 (0.4) | -0.1 (3.6) | -0.1 (3.6) | -0.1 (2.2) | -2.3 (11.5) | 12.1 (72.5) | 3.5 (29.6) |
|  | Therapanacea | -1.0 (3.4) | 0.6 (7.2) | 1.2 (7.6) | 0.1 (2.0) | -0.8 (16.8) | 13.0 (72.5) | 6.5 (30.1) |
